# Supplementary material for: Effect of Speed Threshold Approaches for Evaluation of External Load in Male Basketball Players
Source: Sensors (Basel). 2025 Oct 2;25(19):6085. doi: 10.3390/s25196085 (PMC12526517; doi:10.3390/s25196085)
Supplement: Supplementary file 1 [file sensors-25-06085-s001.zip › sensors-3765570-supplementary.pdf]

Table S1. Glossary of key concepts and technological terms used in the study. Definitions are adapted to the context of basketball external load monitoring to ensure clarity and reproducibility.

Term	Definition
**External Load**	The physical work performed by the player during training or competition, typically quantified through tracking systems.
**Training Intensity**	The amount of physical work performed per unit of time; fundamental for establishing thresholds of physical demands.
**Arbitrary Thresholds or Speed Zones**	Predefined fixed values used to classify speed zones (e.g., >18 km·h⁻¹ as high-speed running), applied equally to all players regardless of individual differences.
**Relative Thresholds or Speed Zones**	Individualized speed zones based on each player's physical capacity. They can be derived from different methods such as maximal aerobic speed (MAS), maximal sprint speed (MSS), anaerobic speed reserve (ASR), or statistical approaches (e.g., k-means clustering). These thresholds provide a tailored representation of external demands.
**Speed Profile**	Classification of players into "fast" or "slow" locomotor groups based on statistical clustering of speed-related variables, as applied in the present study.
**High-Speed Running**	Running efforts typically defined as speeds greater than ~18 km·h⁻¹, an arbitrary criterion established in the basketball literature for male players and commonly used as a benchmark for high-intensity actions.
**Sprinting**	Running efforts defined as events exceeding 24 km·h⁻¹, an arbitrary criterion established in the basketball literature for male players, and widely adopted as a threshold for maximal-intensity actions.
**Seasonal Stages**	Subdivisions of the competitive season (e.g., Stage 1, Stage 2, and Stage 3) used in this study to analyze potential variations in external load distribution across different phases of the season.

Note: The glossary is intended to align with recent methodological recommendations and research in basketball, supporting transparency and comparability across studies.

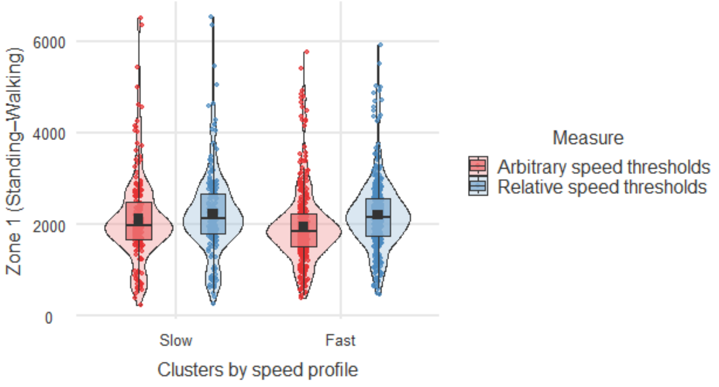

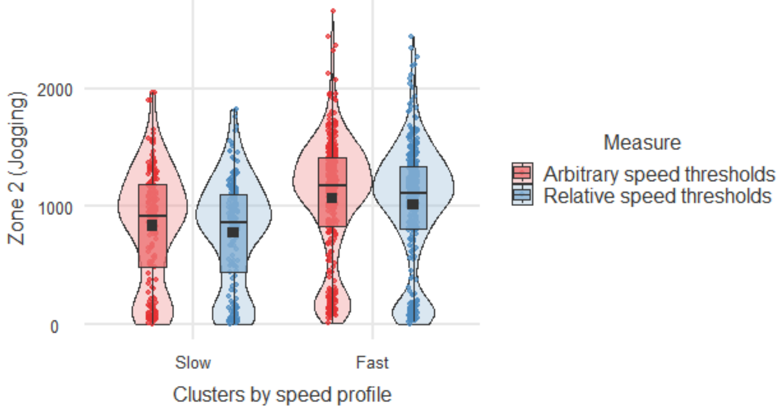

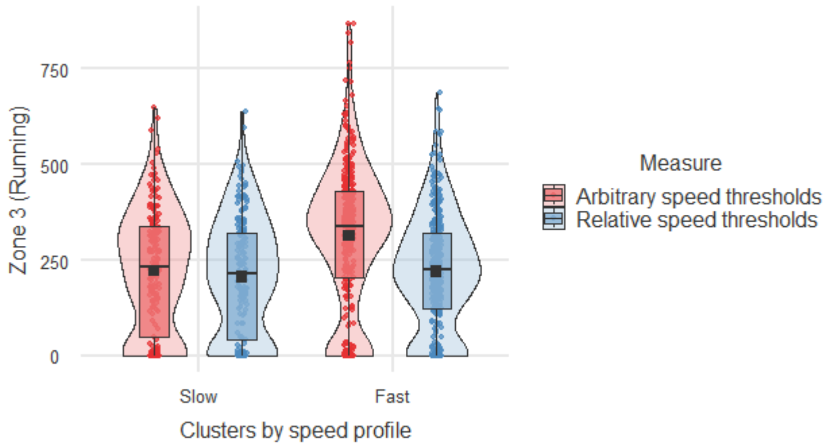

.

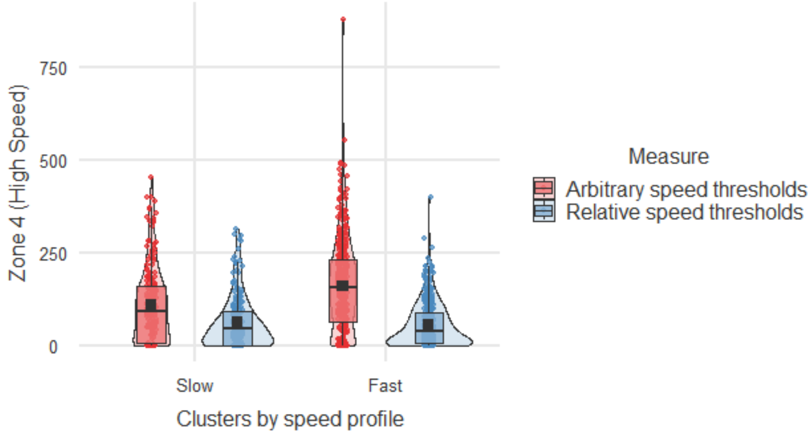

Figure S1. Distance covered across speed zones (Z1–Z4) by players classified as slow or fast, comparing arbitrary (red) and relative (blue) thresholds. Violin plots represent medians, interquartile ranges, and individual distributions.

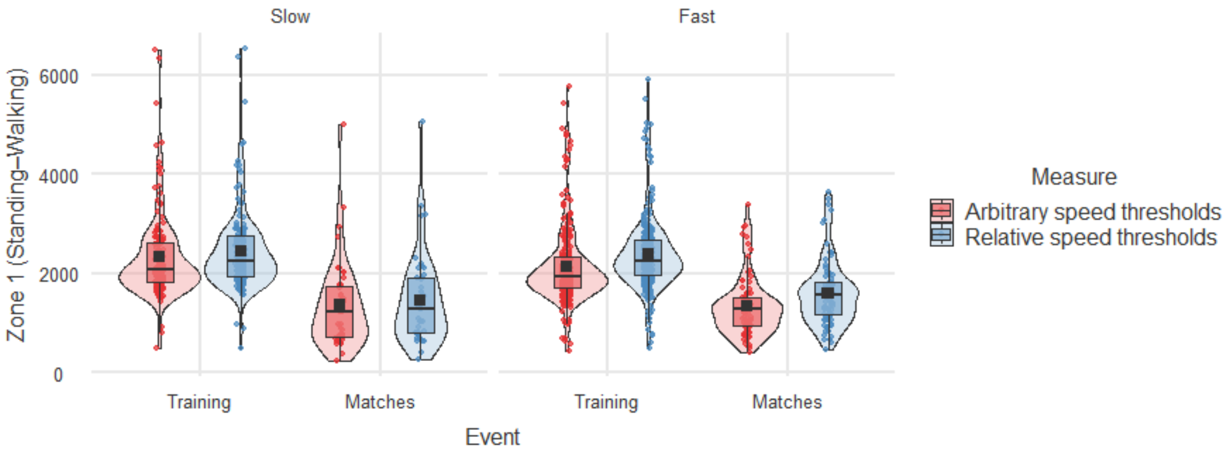

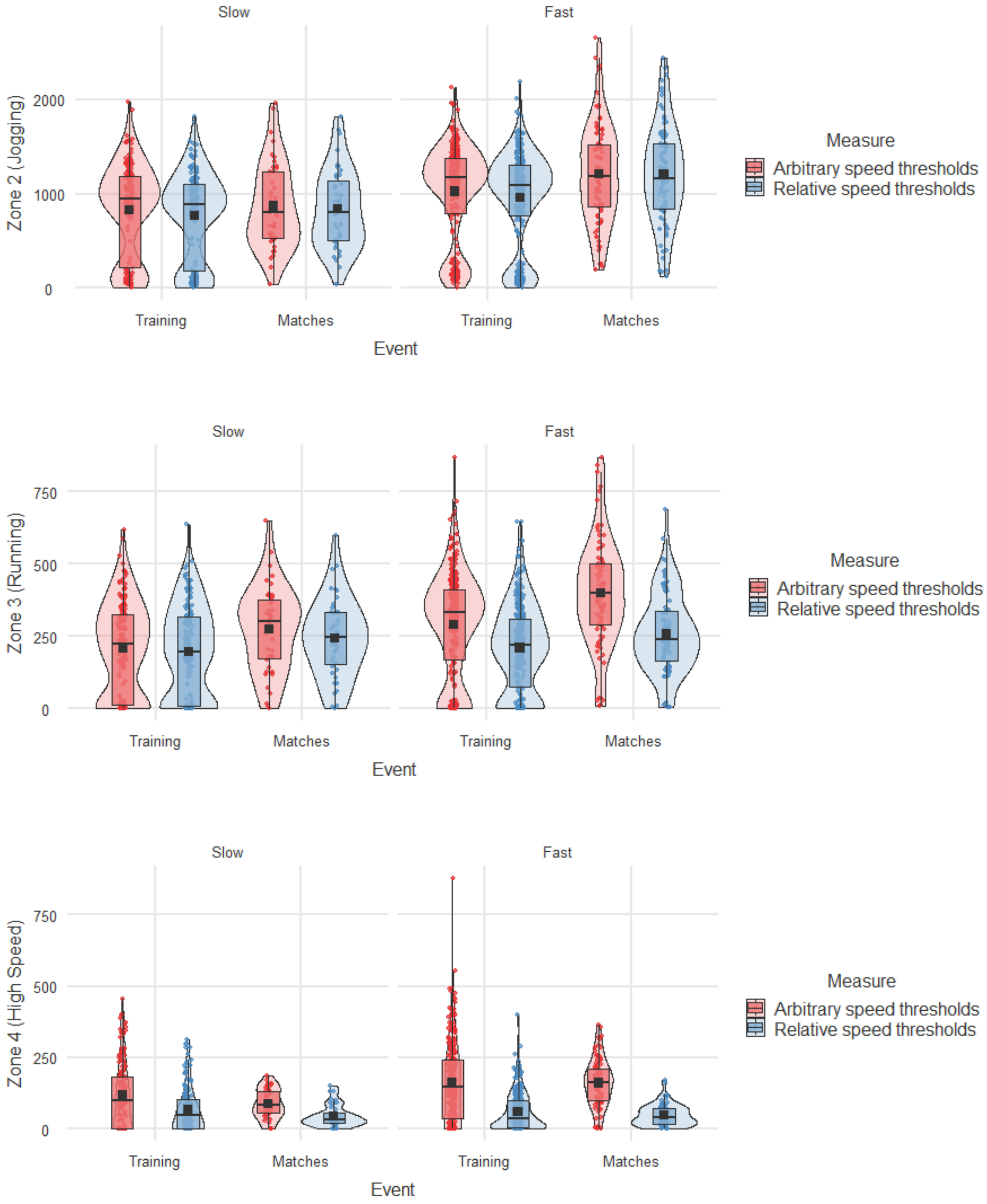

Figure S2. Distance distribution across speed zones (Z1–Z4) during training sessions and matches for slow and fast players, comparing arbitrary (red) and relative (blue) thresholds. Violin plots represent medians, interquartile ranges, and individual distributions.

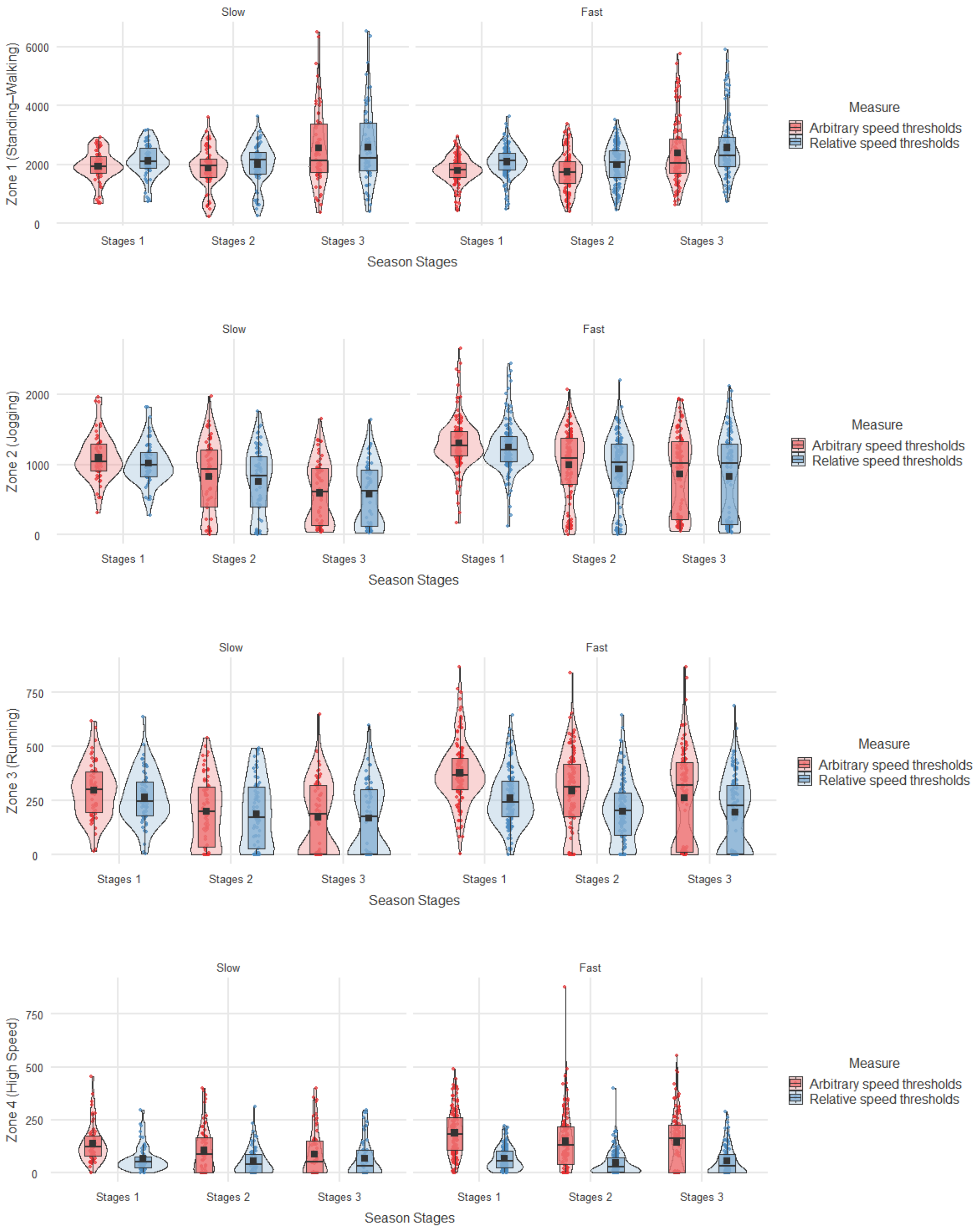

Figure S3. Season stages. External load across season stages (Stage 1, Stage 2, Stage 3) using arbitrary (red) and relative (blue) thresholds. Data are presented as violin plots with medians, interquartile ranges, and distributions.
